# Direct measurements of magnetic interaction-induced cross-correlations of two microparticles in Brownian motion

**DOI:** 10.1038/srep10491

**Published:** 2015-06-02

**Authors:** Maria N. Romodina, Maria D. Khokhlova, Evgeny V. Lyubin, Andrey A. Fedyanin

**Affiliations:** 1 Faculty of Physics, Lomonosov Moscow State University, Moscow 119991, Russia

## Abstract

The effect of magnetic interactions on the Brownian motion of two magnetic microparticles is investigated. The cross-correlations of the thermal fluctuations of the two magnetic microbeads are directly measured using double-trap optical tweezers. It is experimentally demonstrated that the cross-correlation function is governed by the gradient of the magnetic force between the microparticles. The magnetic forces are measured with femtonewton precision, and the magnetic dipole moments of individual microparticles are determined within an accuracy on the order of fA-m^2^.

Brownian motion is the driving force for a wide variety of biological, chemical and physical processes, such as the diffusion of organelles in living cells^1^, the dynamics of DNA supercoils 2,3, and colloidal crystallization^4^. The interactions of Brownian particles play a key role in the collective dynamics of the particles and can induce disorder-to-order transitions in soft matter systems^5^,^6^,^7^,^8^. They govern amazing phenomena, such as the synchronization of particles’ motion 9,10, the self-organization of complex structures from particles^11^ and even the motion of macromolecules 12,13. The interaction of Brownian particles also defines the rheological properties of suspensions, the kinetics of aggregation and phase separation and other colloidal phenomena^14^,^15^.

Optical tweezers have emerged as a unique technique allowing for the precise manipulation of the positions of microparticles, thereby making the distance between interacting particles a controllable parameter. Furthermore, optical tweezers are a powerful tool for directly measuring the interaction forces between microobjects^16^, which provides the possibility of observing the hydrodynamic coupling of two individual optically trapped microbeads, determining the time-delayed anticorrelation of their Brownian motion^17^,^18^ and confirming the results of theoretical studies on the hydrodynamic interaction between microspheres^19^,^20^,^21^. Optical tweezers have also been applied to observe the effects of optical coupling^22^ and electrostatic interactions^23^ on the correlations between microparticles in Brownian motion. The application of an external magnetic field changes the interaction forces between optically trapped magnetic microparticles, allows for the coupling of the particles’ Brownian motion to be controlled, and provides the opportunity to determine the magnetization of individual microbeads^24^. However, the effect of the magnetic interactions of microparticles on the correlations between their Brownian motion remains a challenging, fundamental problem.

In this paper, we describe the effect of magnetic forces between two individual magnetic microparticles on their Brownian motion, observed by utilizing double optical tweezers. Correlations in Brownian motion were measured as functions of the interparticle distance and the external magnetic field strength. It is shown that the coupling of the particles’ motion and the cross-correlations of the fluctuations in particle position in optical traps depend on the gradient of the magnetic force rather than on the value of the interaction force itself. The magnetic moments of individual particles were obtained with an accuracy of several fA-m^2^.

## Theory

**Figure 1:**
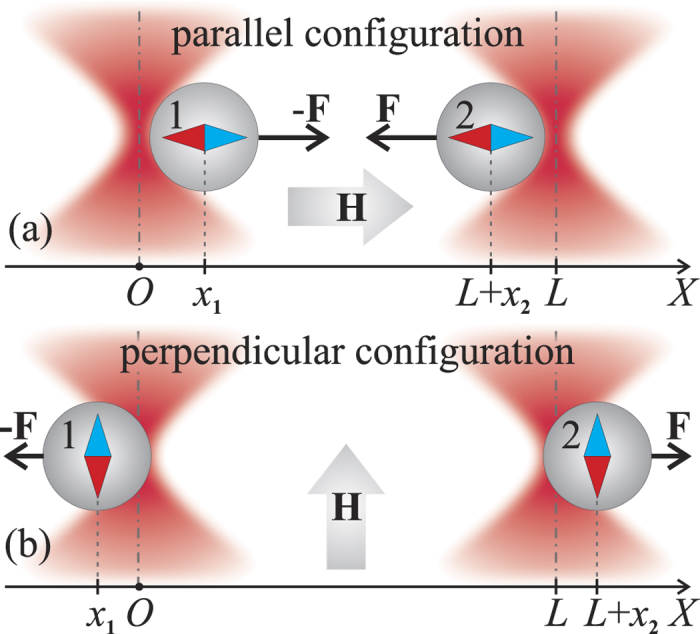
Schematic of optical trapping of two magnetic microbeads in an external magnetic field. Mutual configurations of the magnetic moments of the microbeads (shown as arrows inside the beads) induced by an external magnetic field 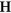: (**a**) parallel to the magnetic force 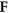 and (**b**) perpendicular to the magnetic force. 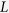 is the distance between the optical traps, and 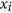 is the displacement of the 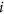-th particle from the center of corresponding trap.

A conceptual schematic of an experiment involving the correlated motion of two magnetic microparticles in an external magnetic field is shown in Fig. 1. Two identical microbeads sharing a magnetic interaction are placed in two optical traps separated by a distance 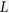. The 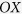 axis is drawn through the centers of the traps. Consider the motion of the particles along the 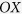 axis, and neglect the motion along the other directions. Due to magnetic interactions and stochastic Brownian forces, the positions of the microbeads fluctuate over time and are shifted from the centers of the optical traps by distances of 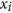 (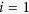, 2). The instantaneous distance between the microparticles is therefore equal to 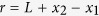. In our assumption 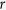 is the length of the vector 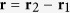, where 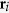 are the positions of the mictoparticles’ centers. The displacements of the microparticles from the centers of the optical traps can be written as 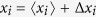, where 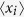 are the mean displacements averaged over time and correspond to the shift in the equilibrium positions of the particles due to magnetic interactions, and 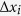 represent fluctuations in the positions of the particles due to Brownian motion. The magnetic force at equilibrium position 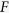 is equal to the restoring optical trap force:
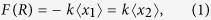
where 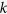 is the effective trap stiffness and 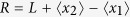 is the distance between the equilibrium positions of the particles. The interaction forces between the particles are equal in magnitude and opposite in direction. Because the microparticles are in Brownian motion around their equilibrium positions in the traps, the fluctuation in the distance between the particles leads to a fluctuation in the magnetic interaction force. In the experiment conducted herein, the fluctuations in particle position were typically two orders of magnitude smaller than the interparticle distance, 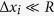 (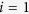, 2), which allowed for the magnetic force to be considered as a following sum of constant and fluctuating components:
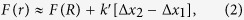
where 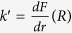 is the gradient of the interaction force.

To describe the motion of the particles in a liquid, we used the approach developed by Meiners and Quake^17^ and Bartlett *et al.*^18^. In this approach the equations of motion for the particles are
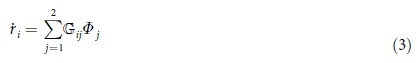
where 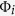 are the total forces acting on the particles. 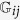 is the Oseen tensor:
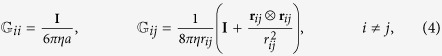
where 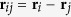, 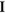 is the 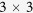 unity matrix, 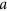 is the radius of the particle, and 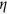 is the viscosity of the surrounding liquid. The Langevin equations for the displacements of the magnetic microparticles along 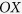 axis, 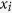, are written as follows:
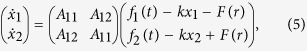
where 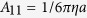, 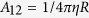 and 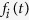 are randomly fluctuating functions with correlation properties 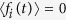, 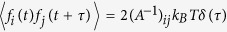. To solve the Langevin equations (5), consider the normal mode coordinates 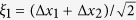 and 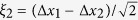, which describe the collective motion of two microspheres and their relative motion, respectively 20. The motion equations for these coordinates are decoupled and can thus be expressed in the following form using Eq. (2):
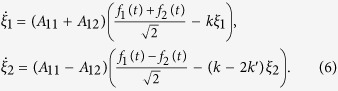


The magnetic interaction between the particles has no effect on the particles’ collective motion mode 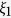. However, the particles’ interaction forces alter the particles’ relative motion mode 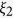. By analogy with Ref. 18, the normalized time cross-correlation function of the particles can be calculated directly using normal motion modes 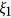 and 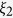:
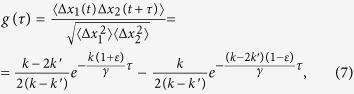
where 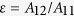, 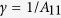 and the dispersions of the particles’ displacements are 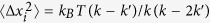. This expression is the central one for the problem to be solved. The correlation function of the magnetic particles’ motion, 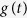, is defined by the trap stiffness 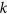 and the interaction force gradient 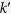 and is independent of the constant part of the magnetic force 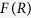 (Eq. 2). Expression (7) is obtained in the general form of a magnetic interaction and remains valid for both dipole and non-dipole magnetic forces. Brownian motion affects the particle-to-particle distance 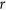, which leads to fluctuations in the interaction force near the 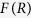 value determined by the force gradient 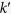. These fluctuations cause the motions of the particles to be coupled; therefore, fitting the experimental cross-correlation function with Eq. (7) allows both the effective trap stiffness 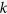 and the magnetic force gradient 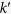 to be extracted.

## Experimental Methods

The experimental setup for the optical trapping of the magnetic microparticles in double optical tweezers is similar to that discussed in detail in Ref. [26]. An oil-immersion objective (Olympus UPLFLN 100XOI2) with a numerical aperture of 1.3 was used to focus the beams of two single-mode diode lasers emitting a wavelength of 980 nm to form the traps. The displacements of the trapped particles were extracted from the signals of quadrant photodiodes, which collected the forward-scattered light of two extra diode laser beams emitting at wavelengths of 635 and 670 nm passing through the sample. Additionally, the setup was combined with a system generating an external magnetic field based on four compact electromagnets. The combined apparatus allowed for two microparticles to be trapped at the desired distance from each other in a homogeneous external magnetic field with a strength of up to 4.9 kA/m, oriented either parallel or perpendicular to the 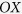 axis. The orientation of the induced magnetic moments of the particles for each case is shown in Fig. 1.

The sample was an aqueous suspension of magnetic beads with a diameter of 3-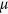
m (PMPEG-3.0, Kisker Biotech GmbH&Co). The microparticles consisted of superparamagnetic magnetite nanograins in a polystyrene matrix. Two beads were optically trapped with an 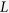 value of several microns at a distance of 20 μm above the surface of a cover glass. The longitudinal displacements of the particles along the 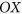 axes in the optical traps were measured with and without an external magnetic field. The means of the particles’ displacements from the traps’ centers and the cross-correlation functions of the particles’ longitudinal displacements from their equilibrium positions were independently measured in each experiment and averaged over five measurements with different pairs of particles.

## Results and Discussion

**Figure 2:**
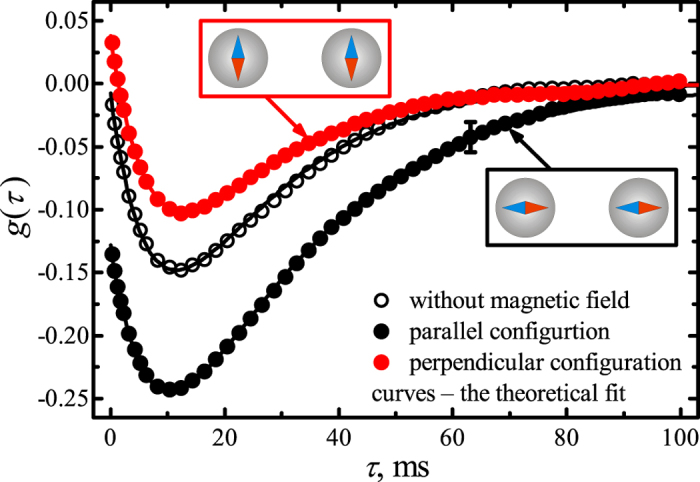
Normalized cross-correlation functions of two magnetic particle displacements as a function of time delay: black and red circles correspond to the magnetic field applied in parallel and perpendicular configurations, respectively, and open circles are data acquired without an external magnetic field; curves are the fit obtained using Eq. (7). The magnetic field strength is 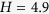 kA/m, and the optical traps’ separation distance is 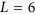 *μ*m.

Typical cross-correlation functions of the particles’ displacements from their equilibrium positions are shown in Fig. 2. All of the functions have a time-delayed minimum at 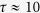 ms, corresponding to the hydrodynamic interaction of microparticles^17^,^18^. The cross-correlation function is significantly modified in the presence of an external magnetic field due to the magnetic moments induced in the particles. For a magnetic field vector parallel to the 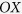 axis, the cross-correlation function decreases, whereas for the perpendicular orientation, 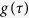 is greater than that in the absence of a magnetic field. In the case of two magnetic moments directed along the 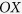 axis (parallel configuration, Fig. 1(a)), the microbeads attract each other; thus, 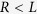. If the magnetic moments are perpendicular to the 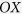 axis (perpendicular configuration, Fig. 1(b)), a repulsive force arises; thus, 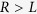. The closer the positions of the particles are, the greater the interaction force becomes. Therefore, in the parallel configuration, the first particle displacement to the right during thermal motion leads to an increase in the attractive force acting on the second particle, and the second particle tends to be displaced to the left, causing a decrease in the cross-correlation function. Conversely, in the perpendicular configuration, the first particle displacement to the right increases the repulsive force acting on the second particle, which will more likely shift to the right. This behavior makes the particles’ movements more highly correlated and causes the cross-correlation function to increase.

The cross-correlation functions are fitted by the theoretical expression Eq. (7) using the magnetic force gradient 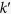 and the effective trap stiffness 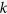 as adjustable parameters; Fig. 2 shows excellent agreement between the model curve and the experimental data. The average value of the trap stiffness is observed to be 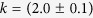 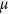
N/m, and 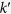 appears to strongly depend on the external magnetic field 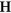 and on the optical traps’ separation distance 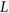.

**Figure 3:**
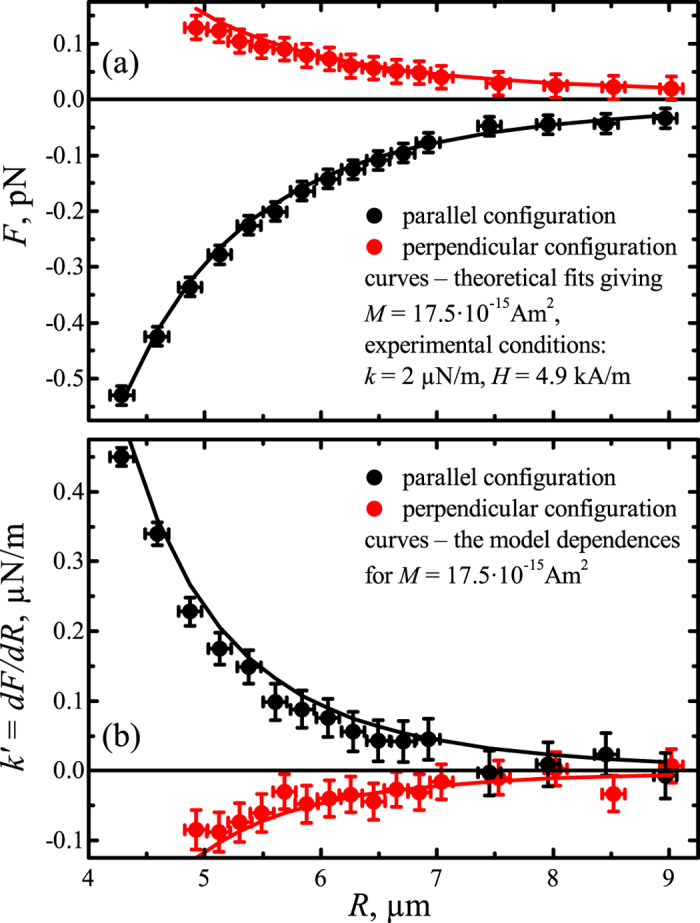
(**a**) Magnetic interaction force (panel a) and magnetic force gradient (panel b) as functions of the distance between magnetic particles obtained from cross-correlation function analysis. Measurements were performed for the parallel orientation (black circles) and perpendicular orientation (red circles) of an external magnetic field with a strength of 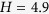 kA/m; curves represent the fits obtained using the model of dipole interactions described by Eq. (10).

The interaction force between magnetic particles, 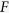, can be determined from the restoring optical force defined, according to Eq. (1), by the trap stiffness 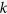 and the particles’ mean displacements from the trap centers, 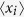. For each value of the distance between the traps, 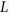, which was varied over the range from 4.5 *μ*m to 9 *μ*m, the particles’ mean displacements 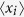 were measured. Then, the average interparticle distance 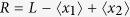 was determined, and the magnetic force 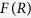 was obtained using Eq. (1); the trap stiffness 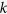 was obtained by cross-correlation analysis. The magnetic interaction force as a function of the distance between the particles is shown in Fig. 3(a). Maximum error of the force owing to transversal displacements under the action of magnetic interaction and the thermal fluctuations do not exceed 1%, which may justify the application of 1D approximation.

When the external magnetic field vector is parallel to the 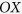 axis (parallel configuration, Fig. 1(a)), the force is positive, indicating an attraction between the particles. For the case in which the magnetic field vector is perpendicular to the 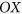 axis (perpendicular configuration, Fig. 1(b)), the magnetic interaction force is negative, indicating that the microbeads repel one another.

The experimental results are interpreted within the model of magnetic dipole interaction between the particles. Each particle is considered to be a dipole with its magnetic moment 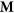 oriented along the external magnetic field vector 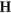. The magnetic force acting on the second particle has the following form^24^,^25^]:
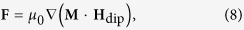
where
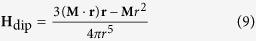
is the magnetic field created by the first particle. For considered experimental arrangements the magnetic force can be expressed as following:
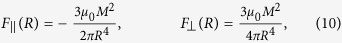
where indices 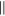 (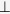) correspond to the parallel (perpendicular) configurations of the applied magnetic field. The fits of the experimental interaction force dependences by Eqs. (10), shown as the curves in Fig. 3(a), yield the microparticles’ magnetic moments in the parallel and perpendicular configurations: 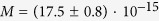 Am^2^ and 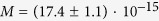 Am^2^, respectively; i.e., in both cases, the magnetic moments are equal within the error indicated by the error bars. In this model, the magnetic moments are assumed to be independent of the distance between the particles because the effect of the mutual magnetization gives a correction to the 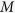 value on the order of 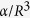, where the magnetic polarizability 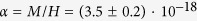 m^3^ is at least twenty times smaller than 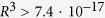 m^3^.

The dependence of the interaction force gradient 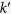 on the distance between the particles extracted from the cross-correlation function analysis is shown in Fig. 3(b). The gradient of the interaction forces in the dipole interaction approximation can be written as follows:
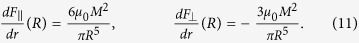


The curves in Fig. 3(b) present the model dependences of the force gradient on the interparticle distance. The dependences were calculated using values of 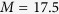
Am^2^ previously obtained from the fitting of the magnetic force. The circles show interaction force gradient obtained using cross-correlation function analysis. The accuracy of the values is approximately 5%, indicating that the method is particularly precise for determining the interaction force gradient, whereas the accuracy of the numerically derived interaction force presented in Fig. 3(a) is approximately 25%.

**Figure 4:**
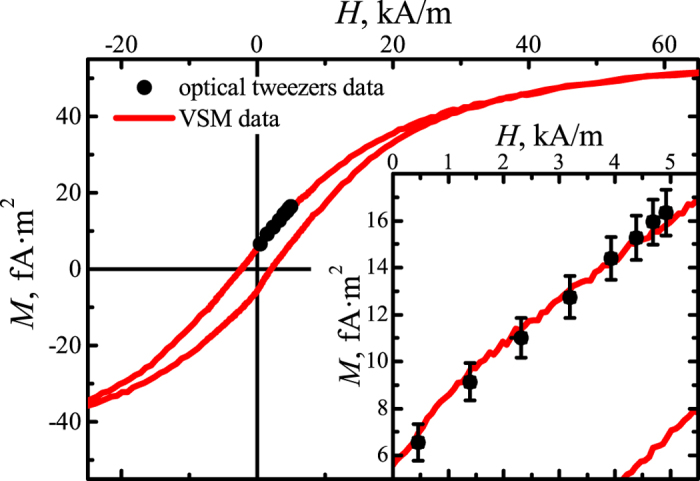
Microparticle magnetic moment as a function of magnetic field measured using optical tweezers (dots) and a vibrating sample magnetometer (curve). Inset: magnification of the data regarding the microparticle magnetic moment.

The dependence of the magnetic moment of individual microparticles on the magnetic field value is shown in Fig. 4. In order to determine magnetic moment the interaction force was measured with the magnetic field decrease from 4.9 to 0 kA/m and the distance between the optical traps fixed to *L* = 6 *μ*m Then the magnetic moment was calculated using Eq. (10). The error bars include the standard error of the mean for 7 experimental realizations (7 different pairs of magnetic particles) and the systematic bias. Vibrating sample magnetometer (VSM) data obtained for the same magnetic microparticle suspension (concentration 50
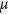
g/ml) were used as a reference and are shown as a curve. The dependence of the mean particles’ magnetic moment on the magnetic field is observed to have a hysteresis loop typical of a superparamagnetic material. The field dependence of the magnetic moments is shown in detail in the inset of Fig. 4 over the range of magnetic field strengths used in the optical tweezers measurements. The agreement between the experimental data obtained using the two different methods illustrates the ability of optical tweezers to characterize magnetic suspensions by the direct measurement of the pair interaction forces and magnetic moments of individual microparticles.

## Conclusions

In conclusion, double-trap optical tweezers combined with a passive microrheology approach were utilized for the direct measurement of the magnetic coupling of two Brownian microparticles. Magnetic interaction leads to correlations between the Brownian displacements of such particles, which are governed by the gradient of the interaction force. The magnetic force as a function of the interparticle distance was experimentally measured with femtonewton accuracy, and the results are supported by theoretical considerations based on the model of dipolar interactions between magnetic microbeads in a viscous liquid. This approach allows for the measurement of the magnetic moments of individual microparticles with a precision of fA-m^2^.

## Additional Information

**How to cite this article**: Romodina, M. N. *et al.* Direct measurements of magnetic interaction-induced cross-correlations of two microparticles in Brownian motion. *Sci. Rep.* **5**, 10491; doi: 10.1038/srep10491 (2015).
